# Development and Validation of Gene-Based SSR Markers in the Genus *Mesembryanthemum*

**DOI:** 10.1155/2023/6624354

**Published:** 2023-10-30

**Authors:** Muhanad Akash, Safwan Shiyab, Mohammed Saleh, Shireen M. Hasan, Mahmoud AbuHussein, Wajdy Al-Awaida

**Affiliations:** ^1^Department of Horticulture and Crop Science, School of Agriculture, The University of Jordan, Amman 11942, Jordan; ^2^Department of Nutrition and Food Technology, School of Agriculture, The University of Jordan, Amman 11942, Jordan; ^3^Hamdi Mango Center for Scientific Research (HMCSR), The University of Jordan, Amman, Jordan; ^4^Higher Council for Science and Technology (HCST), Amman, Jordan; ^5^Department of Biology and Biotechnology, American University of Madaba, Madaba, Jordan

## Abstract

Bioinformatics tools have been employed for the direct development of gene-based simple sequence repeat (SSR) markers. Through the analysis of 28,056 *Mesembryanthemum* expressed sequence tag (EST) sequences, a total of 5,851 ESTs containing SSRs were identified, amounting to approximately 17.07 Mb. Among these, 938 EST sequences harbored more than one SSR marker, and 788 EST-SSR sequences were found in compound form. The most prevalent types of SSR motifs were mononucleotide repeats (MNRs), accounting for 44%, followed by di-nucleotide repeats (DNRs) at 37%, and trinucleotide repeats (TNRs) at 16%. Notably, TNR or longer SSR motifs primarily consisted of shorter repeat lengths, with only 51 motifs containing 10 or more repeats. The BLASTX analysis successfully assigned functions to 4,623 (79%) of the EST sequences. Among the developed primer sets, 21 primers amplified a total of 65 alleles, with primer PMA79 EST-SSR exhibiting the maximum of six alleles. The polymorphic information content (PIC) values ranged from 0 to 0.76, with a mean of 0.47. The marker index (MI) and discriminating power (D) values reached 0.66 (primer PMA63) and 0.95 (primer PMA20), respectively. Utilizing the unweighted pair group method with arithmetic mean (UPGMA), a dendrogram was constructed, successfully segregating the 24 *Mesembryanthemum* genotypes into three distinct clusters, with a similarity coefficient ranging from 0.96 to 0.38. In this study, we have developed a total of 83 EST-SSR primer pairs specific to the *Mesembryanthemum* genus. These newly developed EST-SSRs will serve as valuable tools for researchers, particularly molecular breeders, enabling gene-based identification and trait selection through marker-assisted breeding approaches.

## 1. Introduction

Mesembryanthemoideae (*Aizoaceae*) comprises a single genus, *Mesembryanthemum*, which consists of approximately 101 species and is indigenous to arid and semiarid regions of South Africa [1]. It is also found in the Mediterranean region, the Atlantic Islands, Saudi Arabia, South Australia, and California [2]. *Mesembryanthemum* plays a significant role in its native habitat by thriving in harsh, arid environments where other plants struggle to survive [3]. Several species of *Mesembryanthemum* have been recognized for their antioxidant properties, nutritional and medicinal importance, and ability to accumulate salt, thereby contributing to bioremediation effects [2, 4, 5]. Despite its diverse significance, certain species of *Mesembryanthemum* are classified as endangered or critically endangered by the International Union for Conservation of Nature (IUCN) [6]. Furthermore, molecular research, including the assessment of genetic diversity and genome mapping, has been hindered by the limited availability of codominant molecular markers such as simple sequence repeats (SSRs).

Initially identified in humans, SSRs or microsatellites are repetitive DNA sequences consisting of 1–6 nucleotide core units [7, 8]. These markers are widely distributed throughout most plant genomes. SSR markers possess several advantages, including high variability, codominant inheritance, easy detection, multiallelic nature, transferability between species, and amenability to PCR amplification [7, 9, 10]. However, the development of specific SSR markers typically involves labor-intensive, time-consuming, and costly procedures. The emergence of expressed sequence tag-simple sequence repeats (EST-SSRs) derived from EST and cDNA sequences [11] has become the preferred choice for SSR markers, given the growing availability of EST and cDNA sequences in global sequence databases such as NCBI [12]. Moreover, EST-SSR markers are located in the coding region of the genome, making them ideal DNA markers for cross-species transferability and gene tagging for desired traits [13, 14]. EST-derived SSR markers are expected to exhibit higher conservation and greater abundance among related species compared to anonymous sequence-derived SSR markers [14]. In barley (*Hordeum vulgare* L.), approximately 78% of the 165 EST-SSR markers used successfully amplified in wheat, followed by 75% in rye (*Secale cereale* L.) and 42% in rice (*Oryza sativa* L.) [14].

While EST-SSR markers have been developed and validated for numerous eudicot plants, including *Vicia faba* [15], *Vigna angularis* [16], and *Lens culinaris* Medik [17], to the best of our knowledge, SSR markers have not yet been developed in *Mesembryanthemum*. Therefore, this study was conducted to generate EST-SSR markers specific to the *Mesembryanthemum* genus.

## 2. Materials and Methods

In May 2021, a total of 28,056 *Mesembryanthemum* EST sequences corresponding to 17.07 Mb were retrieved from the National Center for Biotechnology Information (NCBI) website (https://www.ncbi.nlm.nih.gov). These sequences underwent a cleaning process to remove poly-A and poly-T tails using the TRIMEST program sourced from EMBOSS [18]. The identification of EST-SSRs was carried out using the MISA-web program developed by Beier et al. [19]. By employing the MISA-web engine online (https://webblast.ipk-gatersleben.de/misa/), mono, di, tri, tetra, penta, and hexa tandem repeats with minimum repeat unit criteria of 10, 6, 5, 5, 5, and 5, respectively, were selected (Table 1). A total of 7,181 SSR loci were discovered across 5,851 EST sequences. To design EST-SSR primers, the Primer3web software was utilized. The “targets” option was employed to indicate the location of the SSR motif to ensure the selection of appropriate flanking primers. The remaining software settings were maintained as default, except for the annealing temperature (set at 60°C ± 3°C) and primer length (set at 20 bp with a range of +6, −2 bp). A BLASTX search was conducted on the NCBI database to determine the putative function of the developed SSR markers. However, only 28 EST-SSR primers were employed for amplifying the genomic DNA from 24 *Mesembryanthemum* genotypes (Table 2). The iMEC online software [20] was utilized to calculate the polymorphism information content (PIC), heterozygosity index (H), discriminating power (D), marker index (MI), average heterozygosity (av. H), and resolving power (R) for each primer. In addition, a dendrogram representing the 24 *Mesembryanthemum* genotypes was constructed using NTSYS software and the unweighted pair group method with arithmetic mean (UPGMA) [21].

## 3. Results and Discussion

We present the novel development of unique EST-SSR markers derived from easily accessible ESTs for *Mesembryanthemum*. Approximately 17.07 Mb of *Mesembryanthemum* EST sequences, totaling 28,056 sequences, were analyzed to identify 7,181 EST-SSR markers (Table 3). Among these markers, 5,851 ESTs contained a total of 7,181 SSR repeats, indicating that 20.8% of the EST sequences harbored at least one SSR. The frequency of SSR occurrence was calculated as one repeat per 2.38 kb, which is comparable to the frequencies observed in Mentha piperita (1/3.4 kb) and pepper (1/3.8 kb) [22, 23]. Varshney et al. [14] reported that around 5% of ESTs contain SSRs when the minimum repeat length is set to 20 bp, indicating that the frequency of SSRs can vary significantly depending on the search criteria employed. Out of the 5,851 SSRs identified, 938 sequences contained multiple SSRs, and 788 SSRs occurred in compound form (Table 3).

**Figure 1:**
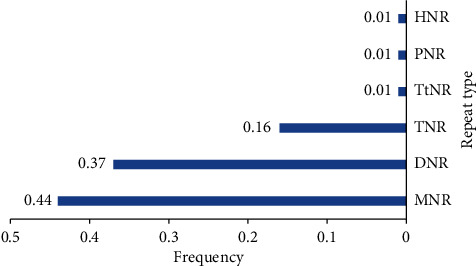
Distribution of different repeat types in *Mesembryanthemum*.

The distribution and frequency of different motifs in SSRs have been observed to vary widely across plant species. In this study, mononucleotide repeats (MNR) were the most abundant (44%), followed by di-nucleotide repeats (37%), and trinucleotide repeats (16%), as depicted in Figure 1. MNRs have been shown to be valuable in bridging gaps in linkage maps constructed using SSR markers [24].

The majority of trinucleotide repeats (TNRs) or longer motifs consisted of shorter repeat lengths, with only 51 motifs containing 10 or more repeats (Table 4). In total, 65 different EST-SSR motifs were identified (Table 1). The most prevalent SSR motifs were A/T (39.2%) for MNRs, AG/CT (32.3%) for di-nucleotide repeats (DNRs), AAG/CTT (3.6%) for trinucleotide repeats (TNRs), AAG/CTT (10.8%) and AAGG/CCTT (0.3%) for tetra-nucleotide repeats (TtNRs), AAGAG/CTCTT (0.2%) for penta-nucleotide repeats (PNRs), and AACAGC/CTGTTG (0.3%) for hexa-nucleotide repeats (HNRs) (Table 1). Similar findings have been reported previously [12, 22, 25, 26]. Considering the increasing percentage of polymorphic markers with longer repeats, only EST-SSRs with 100 bp or more were selected for designing primer pairs. Consequently, 83 primer pairs were developed for *Mesembryanthemum* (Table 5). These SSR markers can be utilized in diversity studies, the construction of genetic linkage maps, and marker-assisted breeding. Furthermore, due to the high transferability of EST-SSRs across species, they can be employed in related species where a limited number of SSRs are available [12, 27].

The BLASTX searches successfully assigned putative functions to 4,623 (79%) of the identified EST-SSRs. This information is valuable for guiding the development of specific markers targeting desired genes and facilitating further exploration of gene-related information [27].

## 4. Validation

Twenty-eight recently designed EST-SSR primers (provided in Table 1) were carefully chosen to encompass all types of nucleotide repeats. These primers were utilized to amplify genomic DNA extracted from 24 *Mesembryanthemum* genotypes. Out of the 22 primers that successfully produced amplification, 21 primers exhibited polymorphic amplification profiles, resulting in a total of 65 alleles being amplified (Table 5). The maximum number of alleles, six in total, was observed for the PMA79 EST-SSR primer. The polymorphic information content (PIC) values, which estimate the discriminatory power of a locus based on allele number and frequencies, ranged from 0 to 0.76, with an average of 0.47 (Table 6). The marker index (MI), which assesses the overall efficiency of a molecular marker, varied from 0 (PMA44) to 0.66 (PMA63), with a mean of 0.41. In addition, the discriminating power (D) of the primers ranged from 0 (PMA44) to 0.95 (PMA20), averaging at 0.67 (Table 6).

**Figure 2:**
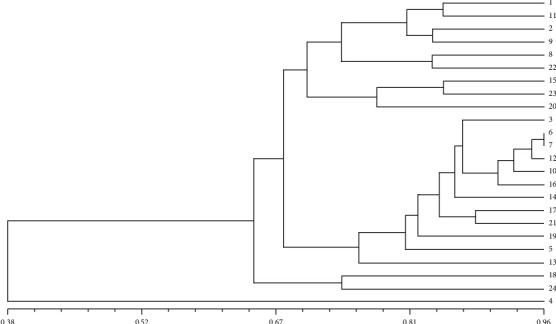
The unweighted pair group method with arithmetic mean- (UPGMA-) based diversity analysis of 24 *Mesembryanthemum* genotypes using 21 expressed sequence tag-simple sequence repeats (EST-SSRs) markers and dice coefficient [28].

The resulting UPGMA dendrogram (Figure 2), which is a visual representation of the genetic relationships, classified the *Mesembryanthemum* genotypes into three distinct clusters. This clustering indicates that there are underlying genetic similarities and differences among the genotypes. The UPGMA method organizes the genotypes based on their genetic profiles, allowing us to observe patterns of relatedness.

The similarity coefficient, ranging from 0.38 to 0.96 with a mean of 0.67, provides a quantitative measure of genetic similarity or dissimilarity among the genotypes. A higher similarity coefficient suggests a closer genetic relationship, indicating that genotypes with coefficients closer to 1.0 share a larger proportion of genetic material.

The diversity in the range of similarity coefficients (0.38 to 0.96) signifies a substantial genetic variation within the *Mesembryanthemum* genotypes being studied. The mean similarity coefficient of 0.67 suggests a moderate level of genetic similarity on average, implying a balanced mix of genetic relatedness and diversity among the genotypes. Understanding the genetic diversity and relationships among these *Mesembryanthemum* genotypes is crucial for various applications, including breeding programs, conservation efforts, and understanding the evolutionary history of these genotypes.

Due to their gene specificity, EST-SSRs are valuable tools for gene tagging and comparative investigations. They can be employed in the development of linkage maps and studies on diversity across related species, as demonstrated by Sahu et al. [27] and Akash and Myers [12]. The newly developed set of EST-SSRs presented in this study offers molecular breeders enhanced resources for gene-based identification and selection of traits through marker-assisted breeding.

## Figures and Tables

**Table 1:** Expressed sequence tag-simple sequence repeats (EST-SSRs) frequencies by repeat motif in *Mesembryanthemum*.

SSR motif	5	6	7	8	9	10	11–20	21–30	31–40	41–50	>50	Total
A/T	0	0	0	0	0	899	1567	257	42	20	28	2813
C/G	0	0	0	0	0	112	202	11	4	0	1	330
AC/GT	0	107	9	6	1	3	4	0	0	0	0	130
AG/CT	0	971	286	191	214	154	386	52	37	14	14	2319
AT/AT	0	134	51	5	3	2	30	7	1	0	0	233
CG/CG	0	2	2	0	0	0	2	0	0	0	0	6
AAC/GTT	64	22	10	9	6	5	0	0	0	0	0	116
AAG/CTT	119	58	45	16	6	7	10	0	0	0	0	261
AAT/ATT	14	130	13	1	1	0	1	0	0	0	0	160
ACC/GGT	71	102	9	29	4	4	0	0	0	0	0	219
ACG/CGT	11	4	3	0	0	0	0	0	0	0	0	18
ACT/AGT	13	3	1	0	0	0	2	0	0	0	0	19
AGC/CTG	31	16	10	25	2	1	0	0	0	0	0	85
AGG/CCT	24	9	8	0	0	1	0	0	0	0	0	42
ATC/ATG	116	39	25	5	17	11	9	0	0	0	0	222
CCG/CGG	31	1	0	0	3	0	0	0	0	0	0	35
AAAC/GTTT	0	0	1	0	0	0	0	0	0	0	0	1
AAAG/CTTT	3	2	0	0	0	0	0	0	0	0	0	5
AAAT/ATTT	0	2	1	0	0	0	0	0	0	0	0	3
AAGG/CCTT	4	5	9	0	0	0	0	0	0	0	0	18
AATC/ATTG	2	0	0	0	0	0	0	0	0	0	0	2
AATT/AATT	2	0	0	0	0	0	0	0	0	0	0	2
ACAT/ATGT	12	0	0	0	0	0	0	0	0	0	0	12
ACTC/AGTG	0	0	3	0	0	0	0	0	0	0	0	3
AGAT/ATCT	0	0	0	0	1	0	0	0	0	0	0	1
AGCC/CTGG	1	0	0	0	0	0	0	0	0	0	0	1
AGGG/CCCT	1	0	0	0	0	0	0	0	0	0	0	1
ATGC/ATGC	0	1	0	0	0	0	0	0	0	0	0	1
AAAAG/CTTTT	9	0	0	0	0	0	0	0	0	0	0	9
AAATC/ATTTG	1	0	0	0	0	0	0	0	0	0	0	1
AAGAG/CTCTT	0	11	0	0	0	0	0	0	0	0	0	11
AATCG/ATTCG	1	0	0	0	0	0	0	0	0	0	0	1
ACAGC/CTGTG	1	0	0	0	0	0	0	0	0	0	0	1
ACCTC/AGGTG	2	0	0	0	0	0	0	0	0	0	0	2
ACTCT/AGAGT	0	1	0	0	0	0	0	0	0	0	0	1
AGAGG/CCTCT	2	0	0	0	0	0	0	0	0	0	0	2
AGGGG/CCCCT	8	0	0	0	0	0	0	0	0	0	0	8
ATATC/ATATG	1	0	0	0	0	0	0	0	0	0	0	1
ATCCG/ATCGG	2	0	0	0	0	0	0	0	0	0	0	2
AAAATC/ATTTTG	0	1	0	0	0	0	0	0	0	0	0	1
AAAAAG/CTTTTT	1	0	0	0	0	0	0	0	0	0	0	1
AAAGAC/CTTTGT	0	1	0	0	0	0	0	0	0	0	0	1
AAAGAG/CTCTTT	1	0	1	0	0	0	0	0	0	0	0	2
AAATGC/ATTTGC	1	0	0	0	0	0	0	0	0	0	0	1
AAATTG/AATTTC	0	3	0	0	0	0	0	0	0	0	0	3
AACACC/GGTGTT	6	0	1	0	0	0	0	0	0	0	0	7
AACAGC/CTGTTG	22	0	0	3	0	0	0	0	0	0	0	25
AACATC/ATGTTG	1	0	0	0	0	0	0	0	0	0	0	1
AACCAC/GGTTGT	1	0	0	0	0	0	0	0	0	0	0	1
AACCAT/ATGGTT	3	0	0	0	0	0	0	0	0	0	0	3
AACTAC/AGTTGT	1	0	1	0	0	0	0	0	0	0	0	2
AACTGC/AGTTGC	2	0	0	0	0	0	0	0	0	0	0	2
AAGAGG/CCTCTT	3	0	0	0	0	0	0	0	0	0	0	3
AAGATG/ATCTTC	2	1	1	0	0	0	0	0	0	0	0	4
AAGGAC/CCTTGT	2	0	0	0	0	0	0	0	0	0	0	2
AATCCC/ATTGGG	1	0	0	0	0	0	0	0	0	0	0	1
AATTAC/AATTGT	1	0	0	0	0	0	0	0	0	0	0	1
ACACAT/ATGTGT	0	2	0	0	0	0	0	0	0	0	0	2
ACAGGG/CCCTGT	1	0	0	0	0	0	0	0	0	0	0	1
ACCCTG/AGGGTC	1	0	0	0	0	0	0	0	0	0	0	1
AGAGAT/ATCTCT	1	0	0	0	0	0	0	0	0	0	0	1
AGAGGG/CCCTCT	0	2	0	0	0	0	0	0	0	0	0	2
AGCAGG/CCTGCT	13	0	0	0	0	0	0	0	0	0	0	13
AGGATG/ATCCTC	1	0	0	0	0	0	0	0	0	0	0	1
AGGGAT/ATCCCT	0	0	2	0	0	0	0	0	0	0	0	2

**Table 2:** The *Mesembryanthemum* genotypes, totaling 24, along with details of their collection sites.

Genotypes	Collection sites	
	Names	Coordinates
1–6	Dead Sea, Jordan	31.3685010, 355447180
7–11	Al-Mudawwara, Jordan	29.2196370, 36.0684550
12–15	Al-Azraq, Jordan	31.7555520, 36.8079137
16–18	Batn Al-Ghoul, Jordan	29.5389940, 35.9398650
19, 20	Alkarama, Jordan	32.0145451, 35.5510075
21, 22	Jordan	—
23, 24	Kingdom of Saudi Arabia	30.5154780, 38.2216491

**Table 3:** Details of expressed sequence tag-simple sequence repeats (EST-SSRs) identified in *Mesembryanthemum*.

Parameters	Numbers
Total number of sequences examined	28056
Total size of examined sequences (bp)	17074324
Total number of identified SSRs	7181
Number of SSR containing sequences	5851
Number of sequences containing more than 1 SSR	938
Number of SSRs present in compound formation	788

**Table 4:** Expressed sequence tag-simple sequence repeats (EST-SSRs) frequencies by nucleotide repeat type in *Mesembryanthemum*.

Nucleotide repeat type	5	6	7	8	9	10	11–20	21–30	31–40	41–50	>50	Total
Mono	—	—	—	—	—	1011	1769	268	46	20	29	3143
Di	—	1214	348	202	218	159	422	59	38	14	14	2688
Tri	494	384	124	85	39	29	22	0	0	0	0	1177
Tetra	25	10	14	0	1	0	0	0	0	0	0	50
Penta	27	12	0	0	0	0	0	0	0	0	0	39
Hexa	65	10	6	3	0	0	0	0	0	0	0	84

**Table 5:** List and characteristics of *Mesembryanthemum* expressed sequence tag-simple sequence repeat (EST-SSR) markers.

Locus name	Primer sequence (5′ ⟶ 3′)	*T*_*m*_ (°C)	Expected size (bp)	GenBank no.	Putative function
PMA01	CCAACAGAACCATCAGCAGC/GCTTCACAAAACCTTACACCCT	59.48/59.04	176	CA834522.1	PREDICTED: transcription factor VIP1 (*Beta vulgaris* subsp. *vulgaris*)
PMA02	AGCATCACATTCAAATCCACTCT/AGAGTCAAGAAGAAAAGGAGGGT	58.40/59.02	189	CA838453.1	No significant similarity found
PMA03	TACAACCAGACCACACACGG/AATCATCATCCAACAAGAAGTGAAT	59.89/57.29	194	AI366624.1	PREDICTED: gibberellin-regulated protein 1 isoform X2 (*Beta vulgaris* subsp. *vulgaris*)
PMA04	TCTATAGACTATCGCGGCCG/AGAGACGGTGAGCACTTTCG	58.63/60.04	246	BE033938.1	No significant similarity found
PMA05^§^	AATGTCGACCACTCTGCTCC/ACTCCAGTAGCTATTGAGTACCA	59.75/58.13	238	AI823067.1	No significant similarity found
PMA06^§^	GCAGAAGTTGATGAAGAAGCCT/TTGATGGGCCGCTACAAGG	58.92/60.08	397	BE033380.1	Hypothetical protein TanjilG_11831 (*Lupinus angustifolius*)
PMA07	TGGGATTGCTTGCTGATCGT/AGAAAGGGCAGCAACTTGGT	60.04/60.11	234	CA840307.1	Uncharacterized protein LOC110732016 isoform X2 (*Chenopodium quinoa*)
PMA08	TACAACCAGACCACACACGG/AATCATCATCCAACAAGAAGTGAAT	59.89/57.29	195	CA834353.1	PREDICTED: gibberellin-regulated protein 1 isoform X2 (*Beta vulgaris* subsp. *vulgaris*)
PMA09	TACAACCAGACCACACACGG/AATCATCATCCAACAAGAAGTGAAT	59.89/57.29	194	BM300798.1	PREDICTED: gibberellin-regulated protein 1 isoform X2 (*Beta vulgaris* subsp. *vulgaris*)
PMA10	TCCTTGATCCGATCTGACTGAC/GGAGAGGGGTGTTTTGGTCA	59.31/59.52	249	BM300496.1	No significant similarity found
PMA11^§^	TACAACCAGACCACACACGG/AGAATCCAATTTTCCAACCGACA	59.89/58.85	167	AW053699.1	PREDICTED: gibberellin-regulated protein 1 isoform X2 (*Beta vulgaris* subsp. *vulgaris*)
PMA12	TGGTGAAGCTTTGATCGAACG/TGTTTTGGTTTCATGGCCCA	59.2/58.5	493	BE034914.1	RecName: full = antimicrobial peptide 1; flags: precursor
PMA13	TACAACCAGACCACACACGG/AATCATCATCCAACAAGAAGTGAAT	59.89/57.29	195	BE033529.1	gibberellin-regulated protein 1-like (*Chenopodium quinoa*)
PMA14^§^	AGTCCTGATCCAATTCGCGG/GGACTACGAGGAGTGTGTGC	60.18/60.11	197	BE033452.1	PREDICTED: Uncharacterized protein At1g03900 (*Beta vulgaris* subsp*. vulgaris*)
PMA15	GCAGCAACCCCACCAACTAA/ACTCACATACCCAAGAATTCCA	60.83/57.07	203	BM300387.1	Similar to mipB gene product in *Mesembryanthemum crystallinum*, encoded by GenBank accession number L36097; MIP homolog; method: conceptual translation supplied by author, partial (*Mesembryanthemum crystallinum*)
PMA16	CCTTCCTTTCTCCATCAGCCA/CTTGGCTAACACCGCAACAC	59.72/60.04	240	BE034350.1	transmembrane 9 superfamily member 2-like (*Chenopodium quinoa*)
PMA17^§^	ACTGATGATCTGTGGTTGTATACA/TCTCCAACACAAAAGAAGATTATGGA	57.46/58.77	214	BE035316.1	No significant similarity found
PMA18	CGATGAGCAGAGGAGAGAGA/GAGGAGGTTTTGTGGTGGGG	58.03/60.54	240	BE036430.1	PREDICTED: 40S ribosomal protein S27-2-like (*Nelumbo nucifera*)
PMA19	AGTTTTCTTTTCCCTCCTCCTCA/GAAATAGGAGCGGGCGAAGA	59.28/59.9	228	BE036273.1	Hypothetical protein M569_05046 (*Genlisea aurea*)
PMA20^§^	TTTCCAATGTCGGTGCTCCA/TGGCGTAACGGATCAAATTTG	59.89/57.52	248	BM301706.1	No significant similarity found
PMA21^§^	TCTAGTAGTGCCCTGCCTGT/TCCCCAATACAACCTTTCCCC	59.96/59.64	222	BE036540.1	24-methylenesterol C-methyltransferase 3 (*Spinacia oleracea*)
PMA22	CGGCTATGTCCTCACCTGTG/AGACGCCTCACAGAACTAGTC	60.18/59.19	244	CA834703.1	RecName: full = ferredoxin-1, chloroplastic; AltName: full = ferredoxin I; flags: precursor
PMA23^§^	CCTGCTTTGTTATTGCTACATCGT/TAAACGCCAGCCAATTGAGG	59.91/58.83	244	BE036370.1	No significant similarity found
PMA24	CCACCTCCAAGTTCTCCCCT/TCGAGTTCTCATCCATGGCG	60.84/59.9	195	CA840662.1	PREDICTED: E3 ubiquitin ligase BIG BROTHER-related isoform X2 (*Theobroma cacao*)
PMA25	CACGGGAAATTGGAGGGTCA/GTGGAAATGTACACGATACGCA	59.96/59.08	332	BE036805.1	Protein SRC2-like (*Chenopodium quinoa*)
PMA26	GGGCATGTATAATTATTCCCCAATGG/GGTCCCCGTTAAAGCCCC	60.13/60.04	557	BE035036.1	No significant similarity found
PMA27	TCCTCCCTCACTCACTTTCAC/CGGGTGGGTGCGAAAGAATA	59.03/60.39	241	DY032226.1	No significant similarity found
PMA28	GACCCGCCAATCTTCTTCCT/TCTGATGGTGCCTTCCTCAC	59.75/59.38	239	BM301459.1	CASP-like protein 4C1 (*Chenopodium quinoa*)
PMA29^§^	TTTTCCACTAAATTTGCACCCTT/AGAGCAATGGAATGTGGAAGTCT	57.38/59.99	250	DR995796.1	PREDICTED: ferredoxin-NADP reductase, leaf isozyme, chloroplastic (*Malus domestica*)
PMA30^§^	TGTGTGGAGCTTGATCAGGG/ATGAGTACTACAGATACCGTTCTTT	59.67/57.36	218	BE035606.1	Hypothetical protein, partial (*Vitis vinifera*)
PMA31	CACTGTGGTTTGCTCTGCTT/GGGGAAAAGGGTGTAATAGAAAGG	58.97/59.05	236	BE034885.1	No significant similarity found
PMA32^§^	CCCGGGCTGCAGGAATTC/ACCCAATTTAGCGGCCACAT	60.83/60.32	236	BE034671.1	No significant similarity found
PMA33	AGTGGAGGGAGAAAGGGGAA/TTTCGTGTTTTACCATAATCCGAA	59.8/57.2	190	CA837391.1	No significant similarity found
PMA34	ACTATTTGATGAAGCACCTGAATG/TCCCAGGCTCCCAATATACCA	57.55/60.06	208	BE577295.1	PREDICTED: zinc finger CCCH domain-containing protein 66 (*Beta vulgaris* subsp*. vulgaris*)
PMA35	TTCCCGTCATCTCCCTCTCT/ACCAATGGCGGAGAAAGTGT	59.37/59.89	239	AI822921.1	PREDICTED: V-type proton ATPase subunit c″2 (*Pyrus x bretschneideri*)
PMA36	AGACATGCAAATTCAATCAACCT/TTTGTAAAAGAGGGCCCGGG	57.27/60.25	258	BE033663.1	No significant similarity found
PMA37	CTCACCTCTTCGTCTCCAGC/ATTCCACCCTCGACGAATCC	59.83/59.54	215	BE035371.1	PREDICTED: mitochondrial uncoupling protein 5 (*Beta vulgaris* subsp*. vulgaris*)
PMA38^§^	TGGAAGACTTGCTCTCTGACT/CCCCTAAATTTTAAAAGCGGCC	58.4/58.47	244	BE036658.1	PREDICTED: uncharacterized protein LOC104896568 (*Beta vulgaris* subsp*. vulgaris*)
PMA39	CACCCTTCCATTCAATGTTCCA/CCTAGATTGAAAGCGAAAGGGG	58.84/59.05	250	BM300354.1	No significant similarity found
PMA40^§^	GCATTTATTCCTTTTCTACATCTTGC/AGATGTGTTTCCGGGTATTCCT	57.65/59.16	227	CA835303.1	chloroplast envelope membrane protein (chloroplast) (*Mesembryanthemum crystallinum*)
PMA41	TCTCTCTCCTCCATTGTTGATTGT/GTGGCCGGAGATATGAGCTG	59.47/60.32	194	BM300809.1	No significant similarity found
PMA42	CCACCAGTCACAATATTACACGC/AGACGAATAGGTTAGTGTGTTCAGT	59.94/59.76	693	BE034183.1	No significant similarity found
PMA43	CTTCCCTCAGGCTGCGAG/AGGGTTAGGCATTGTTGTTGGA	59.81/60.16	247	BE033637.1	PREDICTED: peptidyl-prolyl cis-trans isomerase 1 (*Vigna angularis*)
PMA44^§^	TGCAATATCTGGAAACGCGA/TGAAATGATAGAGAGATCCACCAGG	57.98/59.69	297	CA835421.1	chloroplast envelope membrane protein (chloroplast) (*Mesembryanthemum crystallinum*)
PMA45	TCCTGTGCTCATTTTCCTCTGT/TTCCGCCCCTGTCCTAAATG	59.63/59.75	295	BE036291.1	No significant similarity found
PMA46	CTCCGACATGCAGAACTCCC/GCTAGCTGGTCTGGGTTGAA	60.46/59.68	243	BG269304.1	No significant similarity found
PMA47	TCGGGCTGCAGGAATTCG/GGCAAGCAAAACAACAAGGA	60.13/57.69	250	BE034598.1	Hypothetical protein BVRB_5g115740 (*Beta vulgaris* subsp*. vulgaris*)
PMA48^§^	CTGAGTCCCGTTGCTAGACC/GTGTATGACGTTGCGCTCG	59.83/59.66	242	BE033493.1	No significant similarity found
PMA49^§^	CGGGTTCGTCGATAAAAGAAAGA/TCCTTTCTCTGTTGTTGTTTCTCT	59.08/58.25	216	CA837106.1	PREDICTED: tubulin alpha-3 chain-like, partial (*Raphanus sativus*)
PMA50	AGGAAACAAAGGAGCGGCTC/GGTTGACCTCCCAATCTCGT	60.61/59.39	244	AW266506.1	BEL1-like homeodomain protein 1 (*Chenopodium quinoa*)
PMA51	ATTGTGTTATGAGCTTATGTGTTCA/TCCTCCTTATATGGGCCAGTTG	57.31/59.29	230	BE035895.1	PREDICTED: peamaclein-like (*Juglans regia*)
PMA52	ACAAACAACAAACAACAAACAACACT/GCCCTTGTTCTTGATGCGGA	59.91/60.96	249	BF479882.1	Hypothetical protein BVRB_3g062460 (*Beta vulgaris* subsp*. vulgaris*)
PMA53	CCCGGGCTGCAGGAATTC/TGACACTCAATCACTCGGCG	60.83/60.39	190	BE033624.1	No significant similarity found
PMA54	CCAATTCAAGAGCCGCACTG/GGTGGAAGGAGAGAGGGTGA	59.83/60.25	347	BE034293.1	gibberellin 2-beta-dioxygenase 2-like (*Chenopodium quinoa*)
PMA55^§^	CAAGCTGGGATAATGGTGTCA/AGGATGGAAGAGAAAGAGAGAAAA	58/57.31	241	BE037070.1	PREDICTED: triosephosphate isomerase, chloroplastic (*Beta vulgaris* subsp*. vulgaris*)
PMA56	GAGATAAGCACCTGGGCCTG/TAATGCTTGGGTGGTGGTGG	60.18/60.25	214	BF480398.1	Hypothetical protein VITISV_000212 (*Vitis vinifera*)
PMA57^§^	TCTGGAACTAGTATGTTGATGGAGT/ATAAGGGGAAATTGGGGCCG	59.04/60.11	242	BE035959.1	No significant similarity found
PMA58	AGGGAGTTTGTATGTCAGCCA/CTCACTAGGCAATGGACCGG	59.01/60.18	239	BE035417.1	No significant similarity found
PMA59	TGGTTGGAGAAGATGGAAGACA/CTCATCAAATCATGCTGCCTCA	59.02/59.05	248	CA840173.1	No significant similarity found
PMA60	TGTTCTAGTCCCGTCCGCAT/AAGGAGGGAAGGAGGGAAGG	60.97/60.25	598	BE034770.1	No significant similarity found
PMA61	CTCTCGCTGCTCCATCCCTT/TGAGTTGGATTGGAAATTAGGGA	61.68/57.47	203	BE577610.1	No significant similarity found
PMA62^§^	CCCGGTGTTGGTGGTAGATAG/TGCCGAGAAATAGGAAAGAACA	59.86/57.72	343	BE035340.1	No significant similarity found
PMA63^§^	TCGACCTCCATCTCCACTGT/TTTTACCCAAGAGCCGAACG	59.96/58.48	823	BE033728.1	No significant similarity found
PMA64^§^	CAGAATCCATCCCATACTTCCCA/AGGATAATGATGATTTTGGGTTTGGA	59.6/59.1	212	BM302080.1	Uncharacterized protein LOC110734179 (*Chenopodium quinoa*)
PMA65	AGAAATTATTGGAAATTGTTTGGCC/CAATCTGAAGGGAGAGCGCC	57.31/60.81	208	BE036649.1	No significant similarity found
PMA66	TTTGGGCTGCTTCTGGGATT/ACCACACCTTCATTTACACCC	59.89/58.13	487	BE036567.1	No significant similarity found
PMA67^§^	AGGTCAGAAGGAATCGGCAC/AGTAAATTTGTGTTTTGAAAGGGGA	59.75/57.81	233	BE035280.1	No significant similarity found
PMA68	CCCTCGTTGACACTGGCATA/TCTAGGGGTTTTGGGCGTTG	59.75/60.25	488	BE036046.1	No significant similarity found
PMA69^§^	TGTGGTGCAATTCTCTCATTCA/TGTTCTGTCCCGGCCTTTT	58.25/59.47	300	BE036153.1	No significant similarity found
PMA70	AGAGCAGCTGAACCAACCAA/TTGGAATTCTGCATCTGGGC	59.82/58.52	284	CA840329.1	PREDICTED: probable protein phosphatase 2C 27 (*Beta vulgaris* subsp*. vulgaris*)
PMA71	GGAAGAGGGATGTTCGCCAA/GGGAAAATTTGGGGAAGGGG	60.04/58.71	359	BE034955.1	No significant similarity found
PMA72	CCTCTGCCAATCTTCTTCCCC/GCAGCTTGAGGGAGAGAGAA	60.41/59.1	244	BF479953.1	No significant similarity found
PMA73	AATCCATGGCCTTGTGAAGC/TGTGTTTTGGGAGGGGTGTT	58.81/59.66	297	BE033627.1	No significant similarity found
PMA74	CTTCTAGCTGCAGGAATTCGG/GCAAAGAAACACACCCTCCG	58.79/59.69	287	BE037450.1	Salt-inducible zinc finger 1 (*Sesuvium portulacastrum*)
PMA75	GCCGAATTTGGGACGAGG/AAAGGAGACCGCCCCGAG	58.5/61.39	250	BE035632.1	No significant similarity found
PMA76^§^	GGAGGAGAGGAGAGGAGAGG/AATTGGGCGCGGGAAATAAC	59.52/59.54	400	BE035976.1	Hypothetical protein M569_05046, partial (*Genlisea aurea*)
PMA77^§^	AAACCCTTTTACCACCCAAATC/GGACTTTCCCGGTGGTTGG	57.03/60.6	494	BE036766.1	No significant similarity found
PMA78	CCAGGCCTTAAGATGCTGCA/CAAAAGCACACGGGGAACAA	60.39/59.54	393	BE036806.1	Polyadenylate-binding protein (*Medicago truncatula*)
PMA79^§^	GACTGAAGTAATTCGCGGCC/CGCGCTTTGTGTTTCTCTCC	59.35/60.11	398	BE033686.1	No significant similarity found
PMA80^§^	ATAGGTCTGCAGGCATTCG/GGGTTAAGGGGCGCGTAA	57.31/59.73	460	BE034828.1	No significant similarity found
PMA81	AACGCTCATCTCCTGCTGTC/TTGGTGGTTGTGTTGGGGTT	60.11/60.25	500	BE033954.1	No significant similarity found
PMA82	TCCCACGACTGCAGGAATTC/CTAGCGGGGTATGAGTGAGC	60.04/59.68	471	BE033727.1	No significant similarity found
PMA83^§^	ATGCGCCGACACTCCGAG/GCTTTCTGTGCGTGTGTGC	62.5/60.66	600	BE033402.1	No significant similarity found

^§^Selected for diversity analysis.

**Table 6:** Number of alleles, size range of amplified fragments, and polymorphism statistics calculated with iMEC for 24 *Mesembryanthemum* genotypes using 21 expressed sequence tag-simple sequence repeat (EST-SSR) loci.

Locus	Number of alleles	Size range (bp)	Polymorphic information content (PIC)	Heterozygosity index (H)	Average heterozygosity (av. H)	Marker index (MI)	Discriminating power (D)
PMA5	2	850–1100	0.47	0.38	0.38	0.38	0.94
PMA11	5	167–2000	0.43	0.47	0.47	0.47	0.62
PMA20	1	248	0.47	0.38	0.38	0.38	0.95
PMA21	4	400–950	0.51	0.36	0.36	0.36	0.34
PMA23	1	300	0.53	0.16	0.16	0.16	0.16
PMA29	3	250–700	0.47	0.61	0.61	0.61	0.52
PMA30	1	200	0.54	0.08	0.08	0.08	1
PMA32	5	250–750	0.42	0.5	0.5	0.5	0.8
PMA38	1	244	0.53	0.15	0.15	0.15	1
PMA40	2	230–305	0.41	0.5	0.5	0.5	0.73
PMA44	1	297	0.54	0	0	0	0
PMA48	5	350–1000	0.43	0.47	0.47	0.47	0.86
PMA57	4	350–1000	0.48	0.54	0.54	0.54	0.54
PMA62	1	500	0.46	0.47	0.47	0.47	0.49
PMA63	2	120–800	0.46	0.66	0.66	0.66	0.63
PMA64	5	212–900	0.41	0.5	0.5	0.5	0.74
PMA67	4	233–650	0.42	0.48	0.48	0.48	0.84
PMA76	3	150–400	0.43	0.47	0.47	0.47	0.86
PMA77	4	350–750	0.45	0.41	0.41	0.41	0.92
PMA79	6	250–900	0.42	0.5	0.5	0.5	0.78
PMA80	3	600–800	0.44	0.51	0.51	0.51	0.38
PMA83	2	500–600	0.43	0.48	0.48	0.48	0.64

## Data Availability

The data used to support the findings of this study are included within the article.
